# Enhancing Skin Health: By Oral Administration of Natural Compounds and Minerals with Implications to the Dermal Microbiome

**DOI:** 10.3390/ijms19103059

**Published:** 2018-10-07

**Authors:** David L. Vollmer, Virginia A. West, Edwin D. Lephart

**Affiliations:** 1 4Life Research, Scientific Research Division, Sandy, UT 84070, USA; Davidv@4Life.com (D.L.V.); Virginiap@4Life.com (V.A.W.); 2 Department of Physiology, Developmental Biology and The Neuroscience Center, Brigham Young University, Provo, UT 84602, USA

**Keywords:** human skin, oral applications, collagen, ceramide, β-carotene, astaxanthin, coenzyme Q_10_, colostrum, zinc, selenium, microbiome

## Abstract

The history of cosmetics goes back to early Egyptian times for hygiene and health benefits while the history of topical applications that provide a medicinal treatment to combat dermal aging is relatively new. For example, the term cosmeceutical was first coined by Albert Kligman in 1984 to describe topical products that afford both cosmetic and therapeutic benefits. However, beauty comes from the inside. Therefore, for some time scientists have considered how nutrition reflects healthy skin and the aging process. The more recent link between nutrition and skin aging began in earnest around the year 2000 with the demonstrated increase in peer-reviewed scientific journal reports on this topic that included biochemical and molecular mechanisms of action. Thus, the application of: (a) topical administration from outside into the skin and (b) inside by oral consumption of nutritionals to the outer skin layers is now common place and many journal reports exhibit significant improvement for both on a variety of dermal parameters. Therefore, this review covers, where applicable, the history, chemical structure, and sources such as biological and biomedical properties in the skin along with animal and clinical data on the oral applications of: (a) collagen, (b) ceramide, (c) β-carotene, (d) astaxanthin, (e) coenzyme Q_10_, (f) colostrum, (g) zinc, and (h) selenium in their mode of action or function in improving dermal health by various quantified endpoints. Lastly, the importance of the human skin microbiome is briefly discussed in reference to the genomics, measurement, and factors influencing its expression and how it may alter the immune system, various dermal disorders, and potentially be involved in chemoprevention.

## 1. Introduction

Human skin is the largest organ of the body covering approximately 1.5–2.0 square meters and functions as a physical barrier to protect the body from pathogens, chemicals, physical agents, and solar ultraviolet (UV) radiation throughout life [1,2,3]. Additionally, a recent review by Slominski et al., 2018, described how UV light touches the brain and endocrine system through the skin [4]. The stratum corneum composed of 15 to 20 layers of corneocytes (dead cells) containing filamentous keratin in the outermost layer of the epidermis is a formidable barrier [1,5]. The skin layers also provide essential physiological functions including immune defense, free radical detoxifying enzymes, antioxidant molecules, thermoregulation, prevention of excessive water loss, sensory input via mechanoreceptors, and endocrine (production of vitamin D) and metabolic mechanisms to sustain optimal health [1,5,6,7,8]. Lastly, the keratinocytes express a wide array of molecules including cytokines, growth factors, and receptors [5,9]. There are several reviews on human skin aging (intrinsic or chronological and extrinsic or photo) that adequately cover this topic in more detail including the biochemical and molecular mechanisms that have been reported elsewhere [1,2,3,9,10].

### Topical Application of Cosmetics, Cosmeceuticals, and Oral Supplementation via Nutritionals

While the history of cosmetics goes back to early Egyptian times for hygiene and health benefits, the history of topical applications that provide a medicinal treatment to combat dermal aging is relatively new [11,12]. For example, the concept of cosmeceuticals represents the blending of cosmetics and pharmaceuticals. The term cosmeceutical was first coined by Albert Kligman in 1984 to describe topical products that afford both cosmetic and therapeutic benefits [12]. However, beauty comes from the inside, which means, for some time, scientists have considered how nutrition reflects healthy skin and the aging process [12]. The more recent link between nutrition and skin aging began in earnest around the year 2000 with the demonstrated increase in peer-reviewed scientific journal reports on this topic [12]. Thus, the application of: (a) topical administration from outside to inside the skin and (b) the inside by oral consumption of nutritionals to the outer skin layers is now common place and many journal reports exhibit significant improvement for both on a variety of dermal parameters [11,13,14,15,16,17]. This review covers, where applicable, the history, chemical structure and sources, biological and biomedical properties in the skin, and, lastly, animal and clinical data on the oral applications of: (a) collagen, (b) ceramide, (c) β-carotene, (d) astaxanthin, (e) coenzyme Q_10_, (f) colostrum, (g) zinc, and (h) selenium in their mode of action or function in improving skin health and various dermal endpoints. Since the oral application of collagen is relatively new compared to the other topics, this section is presented in more detail. Lastly, the importance of the human skin microbiome is briefly discussed in reference to the genomics, measurement, and factors influencing its expression and how it may alter the immune system, various dermal disorders, and potentially be involved in chemoprevention.

## 2. Collagen

Collagen is the most abundant protein in the human body where it is responsible for structure, stability, and strength especially within the dermal layers [1,2,3,9,18]. The discovery by Wyckoff et al. and Clark et al. in the mid-1930s demonstrated that collagen was composed of a regular structure at the molecular level [19,20]. With regard to aging, the deposition of collagen (and elastin) decreases with chronological-aging (with the passage of time) and particularly with photo-aging (exposure to the sun) [1,2,3,9]. In addition, it can be broken down by hydrolyzing proteins such as matrix metalloproteinases (MMPs), which results in dermal damage and undesirable skin wrinkles [1,2,3]. The cosmetic industry’s focus on enhancing collagen is known to improve the appearance of the skin especially in the facial and neck areas. Collagen is not only used in the cosmetic industry, but it is also used in pharmaceuticals and the beverage, food, and health care sectors driving the growth of collagen’s use worldwide with the current global market estimated in USD at 3.7 billion and an estimated growth to over 6.6 billion by 2025 [21]. 

### 2.1. Structure of Collagen

Collagen provides support to various tissues such as tendons, ligaments, skin, teeth, and many other connective tissue structures. So far, 28 types of collagen have been identified and described that can be grouped into eight families depending on its structure [1,2,3,18,21]. All collagen proteins have a structure based on three helical polypeptide chains with glycine (Gly) occurring every three amino acid residues while proline (Pro) and hydroxyproline (Hyp) make up about 1/6 of the total sequence of collagen [21,22]. The sequence often follows the pattern Gly-Pro-X or Gly-X-Hyp where X may be any of various other amino acid residues [22]. A typical collagen triple-helix structure can have a diameter of 10 to 500 nm, an approximate molecular weight of 285 kDa, and is composed of 1400 amino acids [21]. Collagen is a viscoelastic material with high tensile strength with low immunogenicity where it can be ingested or injected into a foreign body and can be further modified to eliminate any immune response by heat or chemical treatment [1,21].

### 2.2. Sources of Collagen

Collagen can be obtained from animal and vegetable sources with the most common coming from bovine, porcine, human, and marine organisms such as fish scale and fish skin [21]. There are also synthetic sources of collagen that is commercially known as “KOD” to avoid immune problems [21,22]. These recombinant collagens have been produced from mammalian, insect, yeast, and plant cell cultures [21]. Marine sources of collagen, which have advantages over animal sources due to their greater absorption from their low molecular weight, and negligible biological contaminants such as toxins and low inflammatory effects are more feasible for commercial extraction [21,22].

### 2.3. Biological/Biomedical Properties of Collagen

It is known that collagen hydrolysate has several positive biological properties such as antioxidant, antihypertensive, and lipid-lowering activities as well as the established reparative actions in damaged skin [1,2,3,21]. Furthermore, collagen has a dual action in the skin where it first provides the building block components for collagen (and elastin) and, secondly, it binds receptors in fibroblasts located in the dermal layers to stimulate the synthesis of collagen and elastin as well as hyaluronic acid [1,2,3,21].

### 2.4. In Vitro Studies: Collagen Enhances Fibroblasts and Extracellular Matrix Proteins and Decreases Metalloproteinases (MMPs)

Since fibroblasts located in the dermal layers of mammals synthesize and release a variety to extracellular matrix proteins that improve skin health, there has been a focus on this theme where investigators have examined how collagen supplementation in vitro may result in positive changes in skin and skin-related parameters. 

There are several in vitro studies that have been reported where collagen enhanced fibroblast activities, increased collagen levels to improve collagen’s structural properties, and inhibited MMPs. These have been reviewed [1,2,21,22].

In 2018, Zague et al. reported that collagen peptides modulate the metabolism of extracellular matrix proteins by human dermal fibroblasts (in culture) that were derived from sun-protected and sun-exposed body sites [23]. The in vitro collagen hydrolysate treatment increased the dermal matrix precursors along with procollagen type I and collagen type I proteins. The increased levels of collagen were attributed to enhanced biosynthesis of collagens by fibroblasts but also decreased collagen type I metabolism through the inhibition of metalloproteinases (MMP 1 and MMP 2) activities [23]. The authors concluded that food-derived collagen hydrolysates improved skin cells and dermal health by enhancing collagen production and inhibiting the enzymes that break it down.

### 2.5. Oral Administration and Bioavailability of Collagen: GI Absorption, Distribution into the Bloodstream, and Deposition into the Skin

**Figure 1:**
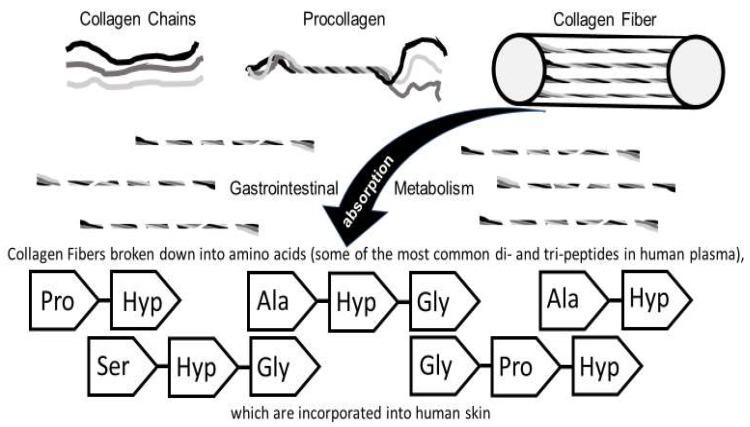
Collagen fiber structure, absorption/metabolism and deposition into skin cells and dermal layers. It is generally thought that collagen (derived products) are hydrolyzed into amino acids in the gastrointestinal tract (GI) prior to being absorbed into the blood circulation, which are then deposited into the skin cells and/or utilized as building block components for extracellular matrix proteins produced by fibroblasts [25,26,27,28]. ALA = Alanine, HYP = Hydroxyproline, GLY = Glycine, PRO = Proline, and Ser = Serine.

In recent years, many nutritional supplements and functional foods containing collagen (peptides, etc.) have been marketed for use in skin care [22]. This section describes how orally ingested collagen (and/or collagen-derived products) via metabolic and absorptive mechanisms have positive effects on skin physiology [22]. The initial studies examined animal models for absorption, transport via the bloodstream, and subsequent distribution or deposition into skin and nails (Figure 1).

### 2.6. Oral Administration of Collagen Improves Skin (and Nail) Parameters in Animal Studies

It is generally thought that collagen (derived products) are hydrolyzed into amino acids in the gastrointestinal tract (GI) prior to being absorbed into the blood circulation (Figure 1). However, increasing evidence suggests that peptides might also be absorbed directly [22,23].

In 1999, Oesser et al., demonstrated that when mice were fed a ^14^C-labeled gelatin hydrolysate, more than 90% of the radioactive tracer was absorbed by the GI tract after 6 h and, at 12 h, the radioactivity reached maximal values in the skin while more than 85% of the radioactivity disappeared from the blood plasma after 24 h [24]. This report showed that peptides with molecular weights ranging from 1 to 10 kDa were absorbed in the GI tract, transferred to the blood, and deposited into cartilage [24]. 

In a porcine animal study in 2006, nine healthy 66-day-old piglets were orally administered porcine skin-derived collagen hydrolysate (0.2g/kg body weight) with a molecular weight range of 3 to 5 kDa for 62 days [29]. Compared to the control group, oral ingestion of collagen hydrolysate resulted in significant increases in both the diameter and density of the collagen fibrils, which suggests that the mechanical properties improved [29].

In 2009, Tanka et al. examined the daily administration of collagen peptides (0.2 g/kg body weight) prepared from fish scales on skin damaged by repeated exposure to UV radiation (VU-B, 03. mW/cm^2^) for six weeks in six-week-old hairless mice [30]. The oral collagen treatment suppressed the UV-B-induced decreases in skin hydration, hyperplasia of the epidermis, and the decreased levels of collagen type I in the skin of the mice [30].

In another animal study of rats, Watanabe-Kamiyama et al. in 2010 examined the administration of 288 mg of chicken feet collagen hydrolysate (800 Da) containing Gly-[^14^C]Pro-Hyp and [^14^C]Pro as radioactive tracers [31]. The radioactivity in the plasma reached a maximal value at 3 h after oral administration along with peak values in the skin at the same time interval. After 14 days of administering this treatment, 70% of the radioactivity was found in the skin while, for the most part, other tissues such as the liver, kidney, spleen, brain, and muscle show decreased radioactivity [31]. 

In 2011, Zague et al. studied how oral collagen hydrolysate administration increased skin collagen expression while, at the same time, it suppressed the activity of the matrix metalloproteinase enzyme (MMP 2) in Wistar rats [32]. The authors suggested that oral collagen hydrolysate administration may reduce skin aging in other mammals.

In 2012, Okawa et al. showed that the oral administration of collagen tripeptide improves dryness and pruritus in the acetone-induced dry skin model in mice [33]. The oral administration of collagen peptides (80 or 500 mg/kg body weight/day) for three days in mice significantly decreased trans-epidermal water loss, suppressed scratching behavior, and normalized axon-guidance factors in the epidermis in addition to reducing pruritus [33].

In 2017, Song et al. examined the effects of collagen hydrolysates from sliver carp skin on UV-induced photo-aging in mice and found that lower molecular weight peptides exerted beneficial effects when compared to high molecular weight collagen hydrolysates on hyaluronic acid levels and moisture content of the skin [34].

In 2017, Chen et al. studied the effect of early enteral nutrition supplemented with Alaska pollock skin-derived collagen peptides on post-burn inflammatory responses in mice [35]. The supplemental treatment decreased NF-κB and TNF-α and IL-6 levels, which suggests that the immune-nutrient supplement may improve post-burn outcomes in burn patients [35].

Lastly, in 2018, Song et al. examined the effects of collagen peptide intake (400 mg/kg body weight/day) for two months in one-year-old female Kunming mice [36]. The results via cytokine array analysis revealed that, in skin, platelet release and a variety of growth factors was inhibited. In plasma, nine cytokines were down-regulated and the authors suggested that collagen peptides may combat cancer and cardiovascular disease via the changes in the quantified parameters [36].

### 2.7. Oral Administration of Collagen Improves Skin (and Nail) Parameters in Human Studies

In 2005, Iwai et al. found that healthy human volunteers who ingested 9–23 g of hydrolysates from porcine skin, chicken feet, and cartilage after 12 h of fasting displayed a maximal concentration of 20–60 nmol/mL in plasma 1 to 2 h after ingestion, which was reduced to half the maximal values at 4 h after ingestion [37]. The porcine skin and chicken feet consisted of collagen type I while the chicken cartilage was collagen type II [37].

In 2006, Matsumoto et al. investigated the effects of daily intake of 7 g of commercially available collagen hydrolysate containing 5 g of fish type I collagen hydrolysates on skin parameters of 25 Japanese female subjects (35 ± 5 years of age) who had dry and rough skin in the winter [38]. After six weeks of treatment, the moisture content of the stratum corneum (face-cheek, forearm, and the back of the neck) increased along with improvement in the pliability and elasticity of the skin, which resulted in greater smoothness, fewer wrinkles, and less skin roughness [38].

In 2007, Ohara et al., in a single-blind crossover study examined five healthy volunteers (33 + 6 years old) who ingested type I collagen hydrolysates (0.385 g/kg body weight) with an average molecular weight of 5 kDa from fish scales, fish skins, and porcine skins after fasting for 12 h [25]. Within 24 h after ingestion, the different hydroxyproline containing peptides in human blood were quantified where fish scales > porcine skins ≥ fish skins, which suggests that the source of collagen provide clues about how these molecules are absorbed and transported into blood [29].

In 2012, Schwartz and Park in a pilot open-label study investigated the effect of a dietary supplement known as BioCell Collagen, which contained hydrolyzed collagen type II, hyaluronic acid, and chondroitin sulfate in 26 healthy females with visible signs of natural and photo-aging in the face [39]. The oral daily supplement of 1 g for 12 weeks led to a significant reduction in skin dryness/scaling and global lines/wrinkles with increased content of hemoglobin and collagen observed, which suggests that the collagen supplement enhanced facial blood (microcirculation) and reduced the signs of aging in the face [39].

In 2014, Proksch et al. conducted a double-blind, placebo-controlled trial examining 69 women aged 35 to 55 years of age that were randomized and received 2.5 or 5.0 g of collagen peptides or placebo once daily for eight weeks (*n* = 23 subjects per group) [40]. At the end of the study, skin elasticity in both collagen groups significantly improved over the placebo group while skin moisture also improved in the collagen groups but did not reach significance over placebo levels. This suggests that oral supplementation of collagen peptides has beneficial effects on skin physiology [40].

Additionally, in 2014, Proksch et al. examined the influence of a specific bioactive collagen peptide VERISOL in 114 women aged 45 to 65 years of age that were randomized to receive 2.5 g VERISOL or placebo (*n* = 57 subjects per group) once daily for eight weeks [41]. By suction blister biopsies, a variety of skin parameters were analyzed. With the ingestion of VERISOL, at four and eight weeks, a significant reduction in eye wrinkle volume occurred and, at eight weeks, significantly higher procollagen type I and elastin levels were quantified, which suggests that oral administration of collagen peptides (via VERISOL) reduced skin wrinkles and skin aging [41]. Notably, this journal article and reported results were reviewed in 2016 [42].

Furthermore, in 2014, Borumand and Sibilla examined, in a clinical study, a nutritional supplement composed of hydrolyzed collagen, hyaluronic acid, vitamins, and minerals (50 mL per day for 60 days), which led to a reduction in skin dryness, wrinkles, and nasolabial fold depth [26]. Additionally, Choi et al., in another clinical study, investigated the effect of collagen tripeptide (3 g/day for four weeks) on wound healing and skin recovery after fractional photo-thermolysis treatment in eight healthy adult volunteers [26]. There were improvements in wound healing parameters in the experimental vs. the controls and the recovery of skin hydration after fractional laser treatment was faster with oral supplementation of the collagen peptides [26].

In 2015, Asserin et al. used two placebo-controlled clinical trials to assess the effect of daily oral supplementation with collagen peptides on skin hydration and collagen density in volunteers [43]. After four weeks of supplementation, dermal collagen deposition significantly increased and, by eight weeks, skin hydration significantly increased. Both enhanced skin parameters persisted after 12 weeks and the ex vivo results showed that collagen peptides induced collagen as well as glycosaminoglycan production [43].

In 2017, Yazaki et al. examined the oral ingestion of collagen hydrolysates in humans (and mice) where the tripeptide collagen was transported into blood with particularly enriched Gly-Pro-Hyp levels that were then deposited into the skin with enriched Pro-Hyp concentrations presumably after hydrolysis [27]. 

In 2017, Hakuta et al. conducted a clinical study of seventeen patients with atopic dermatitis who were randomly assigned to receive a daily (for 12 weeks) 3.9 g of either collagen tripeptide or normal collagen peptides and each subject served as their own control [28]. When their keratinocytes were analyzed, several inflammatory biomarkers were reduced and, in the 13 subjects that completed the study, trans-epidermal water loss was significantly reduced, but the blood parameters were not improved in either treatment group [28].

In 2017, Hexsel et al. performed a clinical study in which they examined an open label, single-center trial of 25 participants who took 2.5 g of VERISOL once daily for 24 weeks followed by a four-week off-therapy period to determine if nail growth and nail health was influenced by the oral VERISOL treatment [44]. The VERISOL treatment increased nail growth by 12% and decreased the frequency of broken nails by 42%. A majority of the participants (80%) agreed that the VERISOL supplement improved their nails’ appearance [44].

Thus, there was an abundance of pre-clinical and clinical reports that provide evidence for collagen hydrolysates supplementation, which supports the enhancement and/or improvement of dermal health via oral administration and via nutritional products.

## 3. Ceramides

In the skin’s epidermis, which consists of the outermost layers, the stratum corneum is the major barrier that functions to: (a) prevent dehydration and water loss, (b) defend against UV radiation and harmful external agents, and (c) maintain dermal health. In addition, it’s an indicator of skin aging [45,46]. In a simplified description, the stratum corneum component organization has two major units: bricks and mortar where corneocytes make up the brick portion of the stratum corneum wall. This is surrounded by a lipid mortar (intercellular “cement”) constituting approximately 20% of the stratum corneum volume that helps maintain the integrity of the tissue elements [28]. The major lipids that form the multi-lamellar barrier of the stratum corneum consists of 50% ceramides, 25% cholesterol, and about 15% fatty acids (by mass) [45,46]. Due to normal desquamation that occurs approximately every 28 days, it is estimated that the skin synthesizes 100–150 mg of lipid per day to replace the lost lipids. Therefore, it represents one of the most active sites of lipid production in the body [45]. Therefore, the coverage of ceramides is important for understanding the function and regulation of the skin barrier and how these lipids play major roles in skin disorders [46].

### 3.1. Ceramides: Structure and Sources

**Figure 2:**
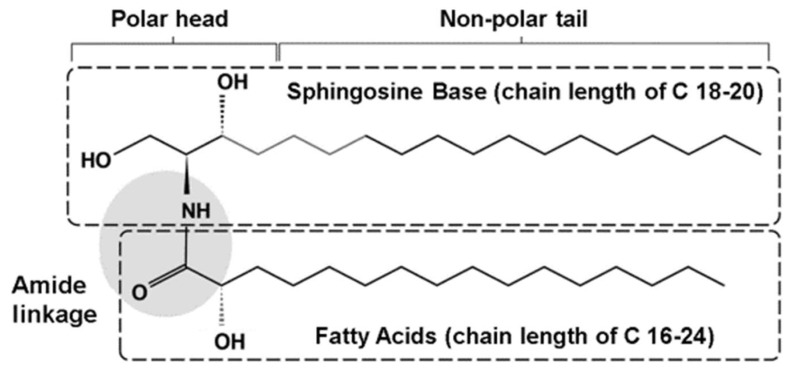
The chemical structure of ceramides. A typical ceramide molecule is composed of long-chain shingosine bases (usually 18 to 20 carbons long) with amide-linked fatty acids (usually 16 to 24 carbons long). The shingonid base contains a polar head and a non-polar tail. In some cases, the fatty acids can be 16 to 36 carbons long [45,46]. The sphinosine base may consist of dihydrosphingosine, sphingosine, phytosphingosine, or hydroxy sphingosine. The fatty acid may be a non-hydroxyl fatty acid, an α-hydroxyl fatty acid, or an esterified ώ-hydroxyl fatty acid. Re-drawn with permission [46].

Ceramides have a basic structure that is composed of a sphingoid base conjugated to a fatty acid via an amide bond (Figure 2) [45,46,47,48]. Ceramides are synthesized in the endoplasmic reticulum of the stratum spinosum, which are then transferred out to create a multi-lamellar barrier between the corneocytes of the stratum corneum [46,48]. There are four different sphingoid bases and three different types of fatty acids that have been described and the number of carbon atoms for each component element varies in length (Figure 2) [46,47,48]. Over 340 ceramide species have been identified within the stratum corneum in human skin [47].

### 3.2. Biological and Biomedical Properties of Ceramides in Skin

Ceramides are the major components of the highly ordered intercellular lamellar structures of membranes and are crucial for the structure and maintenance of skin barrier integrity [46,47,48]. Ceramides are involved in a variety of processes such as cell proliferation and differentiation to apoptosis particularly in lipid raft-mimicking mixtures, human disorders, and diseases [45,46,48,49]. In fact, ceramides have specific structural and signaling roles within the epidermis, which have been reviewed by Uchida et al. in 2015 that include a specific antimicrobial defense [50]. 

### 3.3. Ceramides—In Vitro and Animal Studies

In 2009, Ueda et al. examined the absorption, distribution, and excretion in adult male HWY rats after the oral administration of ^3^H-ceramide [51]. The mean plasma concentration of the ^3^H-ceramide reached maximal levels 10.7 h after ingestion. At 120 h after dosing, the skin to plasma ratio concentration increased by four-fold within the dermis and epidermis. Their results demonstrated that ^3^H-ceramide is distributed gradually into the epidermis after oral administration and gastrointestinal absorption [51]. Additionally, in 2010, Ueda et al. showed that radioactive sphinosine orally administered to mice was absorbed through the digestive tract where it was transported to the dermis and epidermis [52]. It was partly unchanged from its original chemical structure that resulted in enhancing the skin dermal barrier [52].

In 2012, Shimoda et al. examined the changes in ceramides and glucosylceramides (Glc-Cer) in mouse skin and human epidermal equivalents after dosing with an enriched Glc-Cer fraction prepared from rice [53]. In mice, after oral dosing of 3 or 10 mg/kg per day, trans-epidermal water loss (TEWL) was improved and the expression of the ceramide biomarkers was significantly increased. Similar results were obtained in the human epidermal equivalent experiments, which suggested that oral dosing of rice-derived Glc-Cer may improve epidermal water loss and skin barrier function [53].

Lastly, in 2012, Jenneman et al. demonstrated that loss of ceramide synthase 3 caused a lethal skin barrier disruption in knockout mice [54]. The cultured skin from the newborn mice showed that the skin barrier function (due to the loss of long carbon-chain ceramides) was susceptible to the colonization by *Candida albicans* [54].

### 3.4. Ceramides in Human Skin Research

In 2013, Kawamura et al. examined the effects of dried chicken skin powder containing sphingomyelin on human facial skin [55]. In a placebo-controlled, double-blind 12-week study, the treatment was administered at 1 or 2 mg per day in 36 female volunteers who tended to have dry skin. After 12 weeks, skin elasticity showed a significant increase when compared to controls and the results (based upon quantitative endpoints) indicated that ingestion of the dried chicken skin powder containing sphingomyelin improved dry facial skin in the human subjects [55].

There are several human skin disorders and diseases associated with ceramides, which have been reviewed [45,46,47]. These conditions include: (a) psoriasis and lamellar ichthyosis, atopic dermatitis, acne, dandruff, senile xerocis, and the Gaucher disease [45,46,47]. For example, in brief, atopic dermatitis is a common skin disorder affecting up to 18% of the US population, which is characterized by dry skin, pruritus, increased TEWL, and decreased skin barrier function [45,46,47]. It has been established that skin ceramide levels are significantly reduced in atopic dermatitis compared to age-controlled healthy skin [56]. In general, there is a significant decrease in the ceramide levels or alterations in the number of carbons in the ceramide molecule, which is seen in different skin conditions like atopic eczema [47].

Lastly, the influence of skin type, race, sex, and anatomic location on the epidermal barrier function has been reported [57]. In addition, the importance of ceramides in skin and their use as therapeutic agents in skin-care products was reviewed by Meckfessel and Brandt in 2014 [47]. The application of topical, oral, and phyto-derived ceramides has been recently reported along with the implication that ceramide synthases as potential targets for therapeutic intervention in human diseases warrant further research [47,58,59]. The mammalian ceramide synthases were not discovered until the 1990s where the family of six enzymes synthesize ceramides with distinct acyl chain lengths, which have specific tissue-site expression among the synthases [60].

## 4. Carotenoids (β-Carotene)

β-Carotene was first isolated by Wachenroder in 1831 and it was not until 1907 that the empirical formula C_40_H_56_ was reported by Willstatter and Mieg. In 1930–1931, Karrer elucidated the structure of β-carotene [61].

Carotenoids are a class of more than 700 naturally occurring fat-soluble yellow, orange, red, and green leafy pigments synthesized in fruits and vegetables. These carotenoids are divided into provitamin A molecules like β-carotene, α-carotene, and β-cryptoxanthin, which can be converted in the body to retinol and non-provitamin A compounds such as the xanthophylls like lycopene, lutein, and zeaxanthin that cannot be converted to retinol (vitamin A) [62,63,64]. While carotenoids have been shown to exert many mechanisms of action against chronic diseases such as eye disorders, metabolic syndrome, and different types of cancer, this summary will focus on β-carotene only in reference to dermal health [62,63,64].

### 4.1. β-Carotene: Structure and Sources

**Figure 3:**
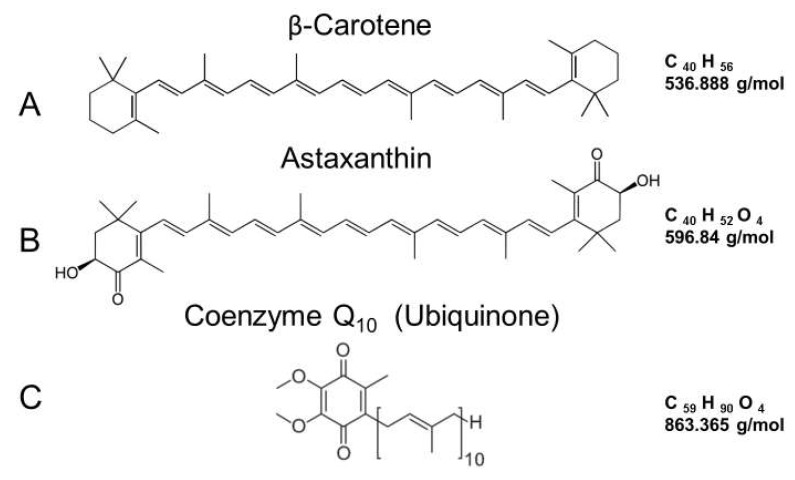
(**A**) The chemical structure and chemical characteristics of β-carotene. β-carotene is a carotenoid found in many naturally occurring fat-soluble yellow, orange, red, and green leafy pigments synthesized in fruits and vegetable [62,63,64]. (**B**) The chemical structure and chemical characteristics of astaxanthin. It is a keto-carotenoid, which is similar in chemical structure to β-carotene and it is found in plants, animals, and algae [67,68,69]. (**C**) The chemical structure and chemical characteristics of coenzyme Q_10_. It is a novel quinone in the lipid extracts of mitochondria and participates in the electron transport chain [70,71].

β-Carotene is composed of 40 carbons (many of which have a linear structure), but it also has β rings at both ends of the molecule with a molecular weight of 536.888 (Figure 3A). Like other carotenoids, β-carotene cannot be produced by the human body and must be taken up by nutritional sources [62,63,64]. The β-carotene content of several selective foods has been reported, which includes carrot juice, pumpkin, spinach, sweet potato, carrots, turnip greens, squash, and cantaloupe. These foods range from 22 to just over 3 mg per 1 cup serving [65]. Commercially available β-carotene nutritional supplements usually contain between 1.5 to 15 mg per capsule [66].

### 4.2. Biological and Biomedical Properties of β-Carotene in Skin

By oral administration, the subsequent absorption of β-carotene into the skin has been reported, which is correlated with dietary intake and bioavailability of the food or the nutritional supplement source [63]. The absorption, metabolism, and transport of β-carotene have been reported elsewhere, but the levels of β-carotene vary in a regional manner within the epidermal and dermal layers dependent upon the body area tested [63,72].

Due to their anti-oxidant properties, β-carotene has been reported to play important roles against oxidative stress and inflammation by its ability to quench singlet oxygen, its ability to be oxidized, and its ability to scavenge free radicals [62,63,64,73]. The oxidative mechanism in skin physiology include reactive oxygen species (ROS), which can be hydroxyl radicals, superoxide, peroxyl and alkoxyl radicals, singlet oxygen, hydrogen peroxide, and organic peroxides [62,63,64,73]. Diets rich in β-carotene or via nutritional supplementation have been shown to prevent cellular damage, premature skin aging, and skin cancer [62,63,64,73]. Specifically, β-carotene has been shown to protect against sunburn (photo-protection) by quenching singlet oxygen, inhibiting free radicals, and suppressing the cellular and tissue response to inflammation [62,63,64].

### 4.3. β-Carotene Skin Research 

A great deal of research on β-carotene in human skin has been reported almost 50 years ago in the 1970s [74,75,76]. The first investigations examined the oral photo-protective effects of β-carotene in erythropoietic protoporphyria [74,75,76]. β-Carotene: (a) is an important vitamin A source for humans, (b) has specific absorption characteristics in humans, (c) is deposited into the epidermis, (d) can be measured by resonance Raman and reflectance spectroscopy, (e) can be converted to retinol, (f) has a specific impact when low versus high doses are utilized, and (g) has safety processes, antioxidant profiles, and signaling pathways, which have been reported [62,63,64,65,66,72,73,77,78,79,80,81,82,83,84,85]. Thus, only select in vitro and in vivo journal reports that examined β-carotene (alone or in some cases in combination with other carotenoids) in human skin are presented below.

### 4.4. In Vitro Studies: β-Carotene in Human Skin 

In 2003, Trekli et al. used the human heme oxygenase 1 (HO-1) gene as a marker of oxidative stress when normal human dermal fibroblasts (FEK4) cells were exposed to UVA irradiation. It has been shown that the induction of this gene occurs via singlet oxygen (^1^O_2_) via UVA radiation and all six all-*trans*-β-carotene concentrations ranging from 0.07 to 21 μM in the culture medium displayed a dose-dependent suppression of UVA-induced transcriptional activation of the HO-1 biomarker [86].

In 2005, Wertz et al. showed that β-carotene interferes with UVA-induced gene expression through multiple pathways in HaCaT human keratinocytes via microarray analysis that include O_2_ quenching along with other gene biomarker mechanisms [87]. Additionally, in 2006, Wertz et al. demonstrated that β-carotene suppressed the UVA induction of matrix metalloproteinases (MMP 1, MMP 2 and MMP-10) that are known to be involved in photo-aging by a singlet oxygen mediated mechanism when HaCaT human keratinocytes were studied [88].

In 2009, Camera et al. reported that β-carotene treatment (along with other carotenoids) in human dermal fibroblasts that were exposed to UVA radiation prevented, in part, the decline in the gene expression of catalase and superoxide dismutase [89]. These results suggested that carotenoids decreased photo-oxidative changes in human dermal cultures.

### 4.5. In Vivo Studies: β-Carotene in Human Skin

Since the 1970s, research on β-carotene in human skin via oral supplementation has been reported with an increase in the number of studies starting in the early 2000s, which has been reviewed elsewhere in 2016 by Pappas et al. and in 2018 by Souyoul et al. [74,75,76,82,90,91].

In 2003, Heinrich et al. reported that oral supplementation of β-carotene (24 mg/day for twelve weeks in subjects with skin type II) significantly increased the carotenoid levels in skin when compared to the control group and protected the β-carotene-treated group from UVA-induced erythema 24 h after the irradiation was administered [92].

In 2008, by utilizing a meta-analysis of published reports up through 2007, Kopckel and Krutmann showed that: (a) β-carotene supplementation protected against sunburn and (b) the study duration had a significant influence on the results where the sunburn protection required a minimum administration of 10 weeks of the β-carotene supplementation [93].

In a study using murine skin, mice were fed a diet supplemented with 0.5% β-carotene for one month and were then exposed to the ozone (0.8 ppm for 6 h per day for 7 days). Valacchi et al., in 2009, showed that the β-carotene dietary treatment downregulated the induction of tumor necrosis factor α, macrophage inflammatory protein 2, inducible nitric oxide synthase, and the oxidative stress biomarker heme-oxygenase-1 (HO-1), which suggested that β-carotene provided protection against ozone-induced pro-inflammatory biomarkers [94].

In 2010, Cho et al. examined the effects of a low-dose (30 mg/day) versus a high dose (90 mg/day) amount of β-carotene for 90 days in 30 healthy female subjects over the age of 50. In only the low dose group, procollagen type I levels significantly increased by 4.4-fold compared to the baseline values and reduced wrinkles and increased elasticity in the photo-aged facial skin of the subjects [81]. Conversely, the high dose of β-carotene had deleterious effects where it increased cutaneous reactivity to UV and UV-induced cutaneous DNA damage. Therefore, this high dose was not recommended [81].

Darvin et al., in 2011, demonstrated that topically applied carotenoids were not as effective as orally administered treatments in 129 healthy female volunteers aged between 21 and 72 years old with skin type II and III [95]. The topically applied carotenoid treatment was stored in the stratum corneum for only a short period of time due to desquamation, tactile contact, washing, and environmental stress while the orally administrated carotenoid treatment was stored in body fat tissue and then slowly released into the skin layers over time [95]. This suggested that a combination of topical and oral applications may represent an optimal form of protection to enrich dermal carotenoid levels.

In 2013, Meinke at al. performed a double-blind placebo controlled clinical study in 24 healthy volunteers where supplementation was given for eight weeks. The results showed the influence of dietary carotenoids, which increased the radical scavenging activity of the skin and provided significant protection against stress-induced radical formation (ROS). The authors also proposed that dietary supplementation could avoid premature skin aging and other associated skin diseases [83].

Thus, the β-carotene has been shown to inhibit free radicals, singlet oxygen-induced lipid peroxidation, decrease pro-inflammatory (ROS-oxidative stress), and aging biomarkers such as MMPs, which protect against extra cellular matrix degradation [90]. Notably, the levels of β-carotene are highest in the summer months, which are associated with the consumption of dietary fruits and vegetables [96]. 

## 5. Astaxanthin 

Astaxanthin was first isolated from lobster by Kuhn and Sorensen in 1938 [97]. It was first commercialized as a pigmentation agent as a feed source in the aquatic-farm industry to increase the orange-red color of the flesh in farm-raised salmonids [67]. Astaxanthin gives salmon and lobster their reddish color and flamingo feathers their pinkish hue [67,68,69]. A great amount of research has been performed examining the effects of astaxanthin due to its wide industrial uses in aqua-farming, food, nutraceuticals, cosmetics, and pharmaceuticals [67,68,69]. 

### 5.1. Astaxanthin: Structure and Sources

Astaxanthin is a keto-carotenoid similar in chemical structure to β-carotene. However, it is not converted into vitamin A [67,96,97] (Figure 3B). Therefore, astaxanthin shares many metabolic and physiological functions attributed to carotenoids [67,68,69]. Additionally, due to the keto-groups and hydroxyl-groups at each end of the astaxanthin molecule, it is more biologically active when compared to zeaxanthin, lutein, and β-carotene [67]. While there are several astaxanthin stereoisomers in nature, the major molecular species in the natural foods, dietary supplement, cosmetic, and food industry appears to be the all-*trans* 3S,3S’astaxanthin. This is derived from chlorophyte algae (*H*. *pluvialis*) since it has the highest capacity to accumulate astaxanthin and safety issues that have been raised concerning the use of synthetic astaxanthin for human consumption [67,69]. The transverse cell membrane orientation of 3S,3S’ astaxanthin has been proposed where the long mid-portion carbon chain intercalates among the hydrophobic fatty acid tails while the terminal keto-groups and hydroxyl-groups are located among the hydrophilic polar heads to provide anti-inflammatory or reduced oxidative stress by electron-conduction along the length of the molecule [98]. 

The sources of astaxanthin include plants, animals, and algae [67,68,69]. For example, astaxanthin is found in several types of seafood including salmon, rainbow trout, shrimp, and lobster. Four ounces of salmon contains about 4.5 mg of astaxanthin [68,69]. Currently, approximately 95% of astaxanthin is produced synthetically via commercial sources due to the cost-effective methods for bulk production. However, the astaxanthin derived from algae sources (*H*. *pluvialis*) have addressed safety issues for human consumption [67]. While there is no recommended daily dosing for astaxanthin, most research studies to date have used between 2 to 24 mg as a daily dosing range [67].

### 5.2. Biological and Biomedical Properties of Astaxanthin

As previously stated, a large amount of research has been conducted on astaxanthin, which has been reviewed [67,68,69]. The most consistent findings in astaxanthin research are the diverse clinical benefits and the potential for anti-aging effects [67,68,69]. From pharmacokinetic studies, absorption of astaxanthin is thought to occur by passive diffusion in enterocytes, which are incorporated into chylomicrons and, then, into low-density lipoproteins (LDL) and high-density lipoproteins (HDL) that are subsequently distributed to tissue sites via the circulation [99,100]. Smoking inhibits astaxanthin’s bioavailability while consumption of a meal with dosing enhances its absorption and bioavailability [100]. One study showed that an oral single 100 mg dose of astaxanthin reached maximum levels at 11.5 h after administration and the plasma astaxanthin elimination time was approximately 21 h [99]. A lower single dose of 10 mg showed that astaxanthin levels reached maximal levels by 6.5 h [99]. 

Astaxanthin as an extremely potent antioxidant was first reported in 1946 by Herisset [101]. Research continued on astaxanthin in the 1950s by Grangaud as a PhD student, but it was not until the 1990s that astaxanthin’s powerful antioxidant properties were becoming widely accepted, which was reported by Miki [102,103]. Since the 1990s, numerous peer-reviewed journal articles have reported on astaxanthin’s health benefits and anti-aging effects in animal and human studies that include: (a) antioxidant [67,68,69], (b) anti-inflammatory [67,68,69], (c) gastro-protective [67,68,69], (d) ethanol and drug-protective [67,68,69], (e) hepato-protective [67,68,69,103], (f) anti-diabetic [67,68,69], (g) cardiovascular-protective [98], (h) anti-cancer [67,68,69,98,99,103], (i) neuro-protective [67,68,69,98,99,103], (j) eye (ocular)-protective [67,68,69,98,99,103], (k) skin-protective [67,68,69,98,99,103], (l) the enhancement of exercise endurance [67,68,69,98,99,103], (m) the enhancement of fertility [67,68,69,98,99,103], and (n) kidney protection against nephrotoxicity [67,68,69,98,99,103]. 

Astaxanthin dosing has demonstrated safety in a number of human clinical trials (up to 40 mg/day). Animal experiments have investigated levels well over 120 mg/day of human equivalents as well as a pigmentation agent or color additive in salmon feeds and as a dietary supplement for human consumption in Europe, Japan, and in the US [69,104,105]. In this regard, the US Food and Drug Administration (USFDA) has approved astaxanthin from *H*. *pluvialis* for human consumption at 12 mg per day and up 24 mg per day for no more than 30 days [106]. Lastly, astaxanthin has been shown to block the 5α-reductase enzyme that converts the principal male androgen and testosterone into dihydrotestosterone (5α-DHT). In this regard, oral administration of astaxanthin has been proposed to be a potential therapy for benign prostatic hyperplasia (BPH), which is dependent upon the potent androgen stimulation via 5α-DHT [107,108,109]. 

### 5.3. Astaxanthin—Skin Research

There are numerous skin studies performed with astaxanthin using topical and oral applications that suggest: (a) prevention of DNA damage and enhanced mitochondrial function and DNA repair through its antioxidant and anti-inflammatory activities via the AKt pathway [67,69], (b) UV protection by decreasing oxidative stress presumably via the NF-κB dependent pathway [67,69,98], (c) immune-enhancing effects by increasing natural killer cell cytotoxic activity [99,100], (d) activation of the Nrf2 pathway, which stimulates the antioxidant defense system to produce more antioxidants [99,100], (e) inhibition of the matrix metalloproteinases (MMPs) that breakdown collagen and elastin [110,111], (f) stimulation of collagen type I and a basic fibroblast growth factor to improve the appearance of wrinkles and promote wound healing [111,112,113,114,115,116,117], and (g) improvement in TEWL, skin texture/smoothness, skin age spots, moisture content, and elasticity [118,119,120,121,122]. The main theme of astaxanthin’s dermal health influences centers around its powerful antioxidant effects [67,68,69,110,111,112,113,117,119,121].

### 5.4. Astaxanthin—Oral Clinical Studies

Since this review is focused on oral applications, only a summary of the total number of clinical studies examining astaxanthin will be covered here listing the human intervention investigations quantifying skin parameters. Astaxanthin in skin health has been recently reviewed in 2018 by Davinelli et al. [67].

In 2006, Yasmashita examined the cosmetic effects on human skin of 4 mg per day of astaxanthin oral supplementation for six weeks in a single blind placebo-controlled study using forty-nine healthy middle-aged women in the US [123]. The obtained results showed significant improvements in fine lines/wrinkles and elasticity by dermatologist’s assessment and moisture content (via a NOVA meter) at the end of the study compared to the initial values of the measured parameters at baseline [123].

In 2010, Park et al. showed that oral administration of 2 or 8 mg of astaxanthin capsules for 8 weeks in healthy female subjects (14 per treatment group) in a double-blind placebo-controlled study resulted in significantly decreased DNA damage, inflammation, and oxidative stress (via quantified biomarkers). It also enhanced the immune response (increased levels in natural killer cells, T cells, B cells, and IL-6) [124].

In 2012, Tominaga et al. performed two human clinical studies. One was an open-label non-controlled study of 30 healthy female subjects where combining 6 mg per day oral supplementation of astaxanthin and 2 mL per day of topical applications of astaxanthin derived from microalgae was given for 8 weeks [111]. At the end of 8 weeks, the subjects displayed significant improvements in the skin wrinkle (crow’s feet), the age spot size, skin texture, and moisture content of the corneocyte layer [111]. In the other study, which was a randomized double-blind placebo-controlled investigation, involved 36 healthy male subjects for six weeks where the dosing was 6 mg of astaxanthin supplementation per day. There were parameters that significantly decreased (wrinkles, TEWL, and sebum oil levels) while, at the same time, skin elasticity and moisture content of the corneocyte layer significantly increased [111].

In 2014, Yoon et al., in a randomized, double-blind placebo-controlled study, examined 44 healthy female subjects who were administered 2 mg of astaxanthin capsules (that also contained hydrolysate collagen) each day for 12 weeks [117]. At the end of the study, in the measured parameters viscoelasticity and procollagen type I levels were significantly increased and TEWL and MMP-1 and MMP-12 were significantly decreased [117]. Notably, it is difficult to portion out the influence of the collagen in the oral capsules and how much influence this factor may have contributed to the measured outcomes. 

In 2017, Chalyk et al. monitored dermal oxidative stress and skin aging biomarkers in 31 middle-aged volunteers (17 men and 14 women over 40 years of age) who were administered 4 mg capsules of astaxanthin for four weeks [125]. Morphological analysis of the residual skin surface components (RSSCs) allowed for detecting age-related changes in corneocyte desquamation and microbiol presence. Blood samples were taken on days 0, 15, and 29 to quantify oxidative stress (biomarkers). The RSSC analysis showed significantly decreased levels of corneocyte desquamation and microbial presence at the end of the study and the blood markers showed that plasma malondialdehyde consistently decreased during astaxanthin administration in the midst of the study, which suggested that astaxanthin produced a strong antioxidant effect resulting in facial skin rejuvenation [125].

Additionally, in 2017, Tominaga et al. conducted a randomized, double-blind, parallel-group, placebo-controlled study using 65 healthy female subjects that were administered either 6 or 12 mg capsules of astaxanthin per day or a placebo for 16 weeks [126]. In both astaxanthin treatment groups, wrinkle formation was decreased compared to baseline values or in the placebo group after 16 weeks. Interleukin-1α levels in the stratum corneum significantly increased in the placebo and low-dose (6 mg astaxanthin) groups, but not in the high-dose (12 mg astaxanthin) group, which suggested that long-term prophylactic astaxanthin supplementation may inhibit age-related skin deterioration [126].

Therefore, the naturally occurring xanthophyll carotenoid astaxanthin originally isolated in 1938 by Kuhn and Sorenson has many anti-aging effects to improve dermal health, which was demonstrated by numerous publications in skin research.

## 6. Coenzyme Q_10_

The discovery of coenzyme Q_10_ began in the mid-1950s [70]. This new compound found to be widely distributed in animal tissues was discovered to be a novel quinone in the lipid extracts of mitochondria and, subsequently, was named coenzyme Q_10._ [70]. Research investigations continued throughout the years and, in the early 1980s, there was an increase in the number of clinical trials examining coenzyme Q_10_. Currently, coenzyme Q_10_ research has been reported to have a positive association with various disorders such as cardiovascular disease and implications to many other conditions like diabetes, cancer, and neurodegenerative disorders [70,71]. 

### 6.1. Coenzyme Q_10_ Structure and Sources

Coenzyme Q_10_ has a chemical name of 2 3-dimethoxy-5-methyl-6-ten-isoprene parabenzoquinone with a molecular weight of 863.365 g/mol [70,71]. Due to having a “tail” of 10 isoprenoid units attached to its benzoquinone “head,” it is named coenzyme Q_10_ (Figure 3C) [70,71]. Its benzoquinone ring with its isoprenoid side group is related in structure to vitamin K and vitamin E [70,71]. It has three redox states, which are ubiquinone, semiquinone, and ubiquinol, and it is found in many cellular/organelle membranes in every cell in the human body [70,71,127]. Coenzyme Q_10_ (Ubiquinone) is the most abundant form in humans and in most mammals. It has been found in plants and microorganisms [128].

All animals including humans can synthesize ubiquinones. Thus, coenzyme Q_10_ is not considered a vitamin and it can be found in human blood (ranging from 0.1 to 0.4 μg/mL plasma from one laboratory or 0.41 to 1.60 μmol/L in men and women from another laboratory) [70,129,130]. Additionally, the distribution and content of ubiquinones in various foods such as meat, poultry, eggs, dietary fats, cereals, dairy products, and fruits and vegetables have been reported [131]. Meats and dietary fats have the highest content ranging from 158 to 16 μg/g fresh weight [131]. Lastly, coenzyme Q_10_ is a very popular dietary supplement that is readily available via commercial nutritional sources. While a recommended daily intake of coenzyme Q_10_ has not been established, nutritional supplement doses range between 30 to 150 mg/day with various formulations that provide rapid delivery into the blood [70,132].

### 6.2. Biological and Biomedical Properties of Coenzyme Q_10_ in Skin

Coenzyme Q_10_ is an endogenous lipid soluble antioxidant present in all membranes, is the cofactor for three mitochondrial enzymes (complexes I, II, and III), and is known to reduce mitochondrial oxidative damage [70,71,133]. The mechanism of action of coenzyme Q_10_ as an antioxidant has been shown to: (a) reduce the production of free radicals [133,134], (b) be involved in the regeneration of vitamin E [71], (c) reduce keratinocyte DNA damage [133,134], (d) reduce UVA-induced MMP production in fibroblasts [135], (e) enhance collagen and elastin expression, inhibit IL-1α, IL-6 production, and melanin synthesis [136,137], and (f) inhibit MMPs and regulate the sulfide oxidation pathway [135,137].

### 6.3. In Vivo and In Vivo (Clinical) Studies: Coenzyme Q_10_ in Human Skin

One of the first reports on coenzyme Q_10_ and human skin in 1999 showed that ubiquinone levels decreased in correspondence with age and UV light exposure. Additionally, in the same report, the authors demonstrated that coenzyme Q_10_ had the efficacy to prevent many of the detrimental effects of photo-aging [138]. Subsequent studies reported during the 2000s demonstrated multiple benefits of coenzyme Q_10_ in both animal models and human skin, which included skin anti-aging properties and, through supplementation, a potential treatment for psoriasis [139,140,141,142]. Additionally, more recent studies from 2015 through 2018 validated the: (a) antioxidant effects of coenzyme Q_10_ to support and maintain cellular energy levels in human keratinocytes [143,144], (b) accelerated regeneration of ATP levels after irradiation in human fibroblasts [144,145], and (c) the safety and effective delivery of coenzyme Q_10_ into the epidermis to improved human skin health [143,144,145]. 

Lastly, one recent clinical study in 2017 examined the effect of dietary supplementation of coenzyme Q_10_ (with either 50 or 150 mg/day for 12 weeks) in a double-blind, placebo-controlled experiment with 33 healthy subjects [146]. Both doses of coenzyme Q_10_ significantly reduced wrinkles and micro-relief lines and improved skin smoothness. Notably, the high dose of coenzyme Q_10_ showed additional improvement of wrinkles in the nasolabial folds, corner of the mouth lines, and upper radial lip lines [146]. However, the coenzyme Q_10_ supplementation did not significantly affect skin hydration or dermal thickness [146]. Therefore, the human skin benefits of coenzyme Q_10_ are well established due to its antioxidant mechanisms of action and involvement in cellular energy levels to maintain homeostasis and enhance dermal health. 

## 7. Colostrum—Source and Composition

Colostrum is the initial milk or “first milk” that is produced by mammals (including humans) immediately following parturition [147,148,149]. The milk fluid produced by all female mammalian species after birth has the function to meet the complete nutritional requirements of the neonate and, at the same time, provide all of the biochemical needs and support the many biological functions of the immature newborn to help the newborn survive and develop [147,148,149]. For example, the development of the newborn gastrointestinal tract and absorption of globulins for immunity are just a few of the functions of colostrum [150,151,152,153].

**Figure 4:**
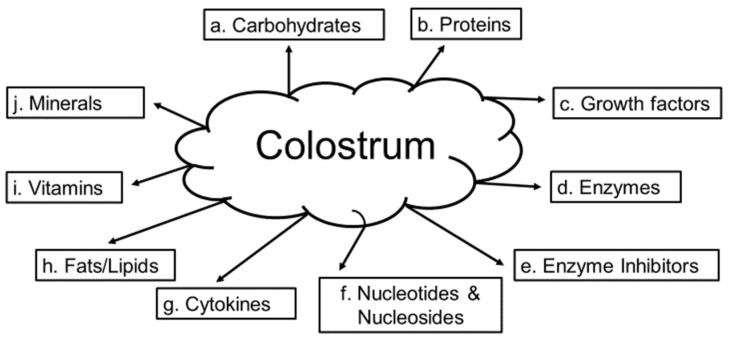
Colostrum or “first milk” is produced by mammals (including humans) immediately following parturition. There are 10 major categories of compounds derived from colostrum, which are shown in the figure above [147,148,149].

The analysis of the chemical composition of bovine colostrum has been reported in detail by McGrath et. al. in 2016 [147]. In addition, human milk composition covering nutrients and bioactive factors are reported elsewhere by Ballard in 2013 [148]. Bovine colostrum is composed of: (a) carbohydrates (where lactose is low, but changes dependent upon the fat and protein content) [147,148,149,150], (b) proteins (casein is higher along with IgG, IgM, and IgA while albumin, lactoferrin, and over 1,000 other proteins have been identified) [147,148,149,150], (c) growth factors (Epidermal Growth Factor (EGF), Insulin Growth Factor IGF), transforming growth factor (TGF), Fibroblast Growth Factor (FGF), platelet derived-growth factor (PDGF) [147,148,149,150], (d) enzymes (70 different enzymes such as antioxidants, proteinases, lipases, and esterases) [145,146,147,148], (e) enzyme inhibitors (e.g., trypsin, α2-antiplasmin, antithrombin, and α2-macroglobulin) [147,148,149,150], (f) nucleotides and nucleosides (pyrimidines and purines) [147,148,149,150], (g) cytokines (interleukins, tumor necrosis factors, and interferons) [147,148,149,150], (h) fats/lipids (fatty acids, phospholipids, campesterol, stigmasterol, β-sitosterol, and cholesterol) [147,148,149,150], (i) vitamins (A, B group, C, E, D, and K) [147,148,149,150,151], and (j) minerals (K^+^, Na^+^, Mg^2+^, Ca^2+^, copper, iron, and zinc) (Figure 4) [147,148,149,150,151].

### Colostrum and Human Skin

There is a lack of journal reports that have examined the influence of colostrum on human skin most likely due to the complex chemical composition of colostrum. However, where data are available, colostrum has several positive influences on human skin. For example, starting in the 1980s and through the mid-1990s, supplemented cell culture medium with milk or colostrum was reported to improve the growth rate of many cell types including skin (fibroblasts) [153,154,155,156]. 

In 2002, Yoon et al. used the known properties of bovine colostrum to promote wound healing. They showed the positive effects of bovine colostrum on human skin where they reported that colostrum did not increase mRNA production of IL-1, IL-6, IL-8, or IL-10 from keratinocytes or human skin biopsy samples, which suggests that dermal healing took place via other mechanisms that may have included growth factors and/or other immune regulatory factors [156].

In 2004, Amiot et al. demonstrated that peptides from milk protein hydrolysates improved the growth of human keratinocytes in culture [157]. The results showed that medium supplemented with 300 μg/mL for 12 days where the average molecular weight of 800 Da containing a high concentration of amino acids promoted the growth of the keratinocytes by 108% [157]. The authors suggested that the mid-size peptides from the milk hydrolysates were metabolized by the keratinocytes that most likely yielded positive growth results.

Lastly, in 2009, Zava et al. compared mare’s milk to colostrum for wound repair function via fibroblastic growth [158]. As expected, colostrum was more effective than milk with the total lipid, linoleic acid, linolenic acid, ganglioside, and glycolipid contents were higher in colostrum when compared to milk. In addition, with further analysis, the fat globule fraction provided the strongest stimulation for wound repair that contained Epidermal Growth Factors [158].

Therefore, while there is not as much information on colostrum and human skin compared to other molecules and factors, the potential for incorporating colostrum fractions into skin formulations is a promising avenue to consider for research and future commercial endeavors.

## 8. Zinc-Essential Mineral Element: Biological, Biomedical Properties and Sources

Trace elements are essential nutrients that your body needs in order to work properly but are needed in much smaller amounts than vitamins and minerals, which are present naturally in foods, added to other foods, and available as a dietary supplement [157,158,159,160,161]. Zinc was discovered by the German chemist Andreas Marggraf in 1746, but it was not until 1963 that zinc deficiency was first recognized in humans [162]. 

A daily intake of Zinc is required because the body has no way to store it long term [159,163]. Zinc is an essential trace mineral involved in: (a) making new cells (cell division) and enzymes that process carbohydrate, fat, and protein in food [159,160,161,163,164], (b) blood clotting and healing of wounds [159,160,161,163,164], (c) immune system function [159,160,161,163,164], (d) protein synthesis [159,160,161,163,164], (e) protecting the function of sensory organs (sight, taste, and smell) [159,160,161,163,164], (f) protecting against oxidative stress and help with DNA repair [159,160,161,162,163,164], and (g) glycemic control (in diabetes) [159,160,161,162,163,164,165]. Good food sources of zinc include: oysters/shellfish, meat, dairy foods, and cereal products such as wheat germ and wholegrain bread [159,161]. The recommended dietary allowances (RDAs) for zinc are 9 to 11 mg for women and men and 11 to 12 mg during pregnancy and lactation [159]. Dietary supplements contain several forms of zinc and the percentage of elemental zinc varies by form. For example, approximately 23% of zinc sulfate consists of elemental zinc. Thus, 220 mg of zinc sulfate contains 50 mg of elemental zinc. Notably, the elemental zinc content appears on the Supplemental Facts panel on the supplement container (in the USA) [159]. It is important to maintain a “steady state” level of zinc since too little or too much may result in physiological dysfunction and/or disease [159,162].

### Zinc and Human Skin

Skin bioavailability of dietary zinc (and selenium) has been reviewed by Richelle in 2006 and Souyoul in 2018 [82,91]. Zinc plays an important role in the three skin functions such as morphogenesis, repair, and maintenance that provide protection and defense via the proteins and enzymes that are involved in these processes [160,161,162,163,164]. Approximately 6% of the total concentration of the body’s zinc is located in the skin, which is present at levels five-fold higher in the epidermis when compared to the dermis [160,161,162,163,164]. Zinc is known to stabilize cell membranes, act as an essential cofactor for several metalloenzymes (MMPs), be involved with superoxide dismutase, metallothionein, DNA, and RNA ploymerases, and participate in basal cell mitosis and differentiation [159,160,161,162,163,164,165,166,167,168].

Zinc and titanium are also common components in topical sunscreens for its ability to block UV light rays [159,168]. Usually, unless the barrier properties of the skin were disrupted (e.g., burn patients) or an extremely high topical application (>40%) was applied, the blood zinc levels were not altered [169,170,171]. Moreover, the risk assessment of zinc oxide used as a UV filter in sunscreens has been reported to be safe [171,172].

In induced (experimental) or clinical deficiency of zinc, skin lesions were common along with delayed wound healing and rough skin [160,162,167,168]. During the early phases of wound healing, the induction of metallothionein may provide a source of zinc required for metalloenzymes to function. Superoxide dismutase and glutathione peroxidase are upregulated following cutaneous injury that, presumably, are zinc-dependent [173,174].

Notably, there are also skin and hair-related symptoms and conditions associated with a zinc overdose while very rare disorders of the skin and appendages include acrodermatitis enteropathica. Acrodermatitis enteropathica is a rare genetic autosomal recessive disorder characterized by periorificial dermatitis, alopecia, and diarrhea. This disorder is caused by mutations in the gene that encodes a membrane protein binding zinc [160,168,175]. Additionally, in 1986, Mulherm et al. showed that excess zinc in the diet of lactating female mice affects pigmentation and the condition of the skin of the off spring [176], which suggests that zinc plays several important roles in skin parameters.

Lastly, the important aspects of zinc have been reviewed within the past four years covering skin biology, skin disorders, wound healing, and clinical applications, which are described in detail elsewhere along with the ZIP2 protein. This protein is a zinc transporter that is associated with keratinocyte differentiation [175,177,178,179,180]. 

## 9. Selenium—Essential Mineral Element: Biological and Biomedical Properties and Sources

Selenium is a trace element (like zinc). It is found in the soil and is present naturally in many foods, added to other food sources, and available as a dietary supplement [181]. Selenium was discovered by Jöns Jacob Berzelius, a Swedish chemist, in 1817 after analyzing an impurity that was a contaminate in sulfuric acid in a factory in Sweden [182]. Notably, the amount of selenium in drinking water is not nutritionally significant in most geographical locations [183]. Selenium has attracted attention because of its antioxidant properties (see below). Selenium exists in two forms: inorganic (selenate and selenite) and organic (selenomethionine and selenocysteine). Both forms can be good dietary sources [181,183]. It is composed of more than two dozen selenoproteins that play important roles in reproduction, thyroid hormone metabolism, DNA synthesis, and protection from oxidative damage and infection [184,185]. Most selenium is in the form of selenomethionine in animal and human tissue [181]. Good food sources of selenium include: Brazil nuts, fish (yellowfin tuna, halibut, sardines), meats, poultry, eggs, grains, and breads, which range from 544 to 13 mcg per serving [181]. The recommended dietary allowances (RDAs) for selenium are 55 mcg in adult men and women and 60 to 70 mcg in pregnancy and during lactation [181]. Dietary supplements contain various amounts and forms of selenium since the human body absorbs more than 90% of selenomethionine but only approximately 50% of selenium from selenite [186]. Selenium is stored in various tissue sites (liver, kidney, skeletal muscle, brain, and testis, etc.) [183,184]. The selenium stored in liver is most labile, which is followed by the kidney and skeletal muscle [183].

Similar to zinc, it is important to maintain adequate levels of selenium since too little or too much may result in physiological dysfunction and/or disease [181,183,184,186]. Selenium deficiency is associated with an increased risk of several types of cancer including skin cancer [187].

### Selenium and Human Skin 

Skin bioavailability of dietary selenium has been reviewed by Richelle in 2006 and Souyoul et al. in 2018 [82,91]. Selenium is present in skin cells that aid in: (a) glutathione peroxidases (GPXs) and thioredoxin reductases as a cofactor (GPX is a good biomarker for selenium levels) [82,91], (b) removing harmful lipid hydroperoxides, hydrogen peroxide, and peroxynitrites [82,91], (c) DNA synthesis and repair [82,91,187], (d) preventing oxidative stress [82,91,187], cell membrane destabilization, and DNA damage [82,91,187,188], and (e) preventing the effects of UVB-radiation and induced apoptosis [82,91,187,188,189]. In 1998, Rafferty et al. showed that there are qualitative and quantitative differences in selenium expression between keratinocytes, melanocytes, and fibroblasts in culture [188]. 

Topically applied *L*-selenomethionine has been reported to reach systemic circulation [190]. With respect to selenium and skin, inadequate selenium does not manifest as skin lesions. However, excessive selenium does. Selenium toxicity is characterized by garlic breath, brittleness and loss of nails and hair, and gross skin lesions [184,185]. These clinical manifestations disappear when the source of excess selenium is reduced or removed. While selenium toxicity is usually rare, a clinical case reported of a 35-year-old man in India that consumed wheat grain, which contained 250-times the normal selenium levels. His case presented with several physiological disorders including skin abnormalities [191]. High selenium levels regionally in the soil have been reported throughout the world [192]. 

It should be mentioned that the “therapeutic window” of the spectrum of tolerable dosing of selenium is relatively narrow where the lowest dose not including symptoms of deficiency and the highest levels not inducing toxicity is between 0.1 to 1.0 μg /g body weight. Thus, this makes it difficult to study selenium as a micro-element in its influence on skin and hair conditions. For example, Hwang et al. showed in 2011 that changes in murine hair with dietary selenium excess or deficiency caused alopecia with an associated significant decrease in the anti-apoptotic Bcl-2 levels and a corresponding increase in the pro-apoptotic BAX levels [193]. 

In this regard, it has been shown that seleno-proteins are essential for proper keratinocyte function, skin development, and wound healing [194,195,196]. Additionally, selenium has been proposed to treat psoriasis since a large-scale study showed that the concentration of selenium in patients with psoriasis was lower when compared to healthy subjects [197,198]. Lastly, selenium has been proposed as a potential treatment for cutaneous melanoma [199].

In 2013, Hazane-Puch et al. examined the importance of the chemical form of selenium used in human skin among in vitro investigations. They examined the effects of the inorganic form of selenium (sodium selenite, Na_2_SeO_3_) and compared this to results obtained by using the organic form of selenium (seleno-*L*-methionine, SeMet) in human keratinocyte (HaCaT) cells that were exposed to UVA radiation [200]. Only the SeMet form protected the HaCaT cells from UVA-induced cell death despite the fact that both selenium forms increased glutathione peroxidase-1(GPX1) activity and selenoprotein-1 (SEPW1) expression. Although both selenium compound forms conferred antioxidant protection, the range of protection was higher with SeMet, which suggests that the chemical form is an important determinant of its biological function as an essential nutrient [200].

In 2014, the same investigators performed additional studies where a six-day selenium supplementation led to either UVA-photoprotection or toxic effects in human fibroblasts in vitro depending upon the chemical form utilized and the dose of selenium [201]. Hazane-Puch et al. showed that, at a 5 μM dose of sodium selenite, cell proliferation was inhibited, which was associated with blockage at the G2 phase and induced DNA fragmentation leading to caspase-3-dependent apoptosis. At low doses, (<1 μM) SeMet and sodium selenite induced GPX1 activity and SEPW1 expression but MMP1 was increased by sodium selenite only [200]. At these low doses, both forms did not protect the human fibroblasts from UVA-induced apoptosis. However, SeMet decreased malondialdehyde (MDA) and protected the fibroblasts from the UVA-induced increases in MMP1 and MMP3. Thus, SeMet apparently may be a potential agent for the prevention and treatment of skin photo-aging [201].

Undoubtedly, there are many beneficial effects of selenium on human skin health involving its antioxidant properties along with various other biochemical actions. Assuredly, this research topic and others will continue to be explored in the future that will resume the story of essential elements and natural compounds, which started with how human nutrition can have applications for the improvement of dermal health and well-being as nutri-cosmetics [82,91,202,203]. 

Lastly, all eight of the natural compounds and minerals covered in this review as far as their involvement in maintaining and improving skin health along with their mechanisms of action are displayed in Table 1.

## 10. Other Selected Natural Compounds and Vitamin D: Effects on Skin Biology

While this review covered animal and clinical data on the oral applications of: (a) collagen, (b) ceramide, (c) β-carotene, (d) astaxanthin, (e) coenzyme Q_10_, (f) colostrum, (g) zinc, and (h) selenium, it is important to note that other natural compounds/molecules have positive influences on human skin health. Some of these notable natural compounds include green tea polyphenols, grape seed proanthocyanidins, silymarin, cocoa polyphenols, and resveratrol along with its polyphenolic-related compounds known as the isoflavonoids including genistein and equol, which have been reported and reviewed elsewhere [3,9,13,16,204,205,206,207,208]. These bioactive food compounds have the ability to improve skin health and reduce skin aging by: (a) acting as antioxidants and stimulating the Nrf2 pathway to generate other antioxidants [3,9,13,16,204,205,206,207,208], (b) stimulating collagen, elastin, and TIMP 1 while, at the same time, inhibiting MMPs and the enzyme elastase [3,9,13,16], (c) inhibiting inflammatory molecules such as the interleukins and cyclo-oxygenase (COX) compounds, and the inhibition or counteracting of ROS formation [3,9,13,16,204,205,206,207,208], (d) inhibiting NFkappB and AP-1 [3,7,11,204,206], (e) stimulating SIRT 1 gene expression (the anti-aging biomarker) [3,9,206], (f) binding of the potent androgen, 5α-DHT, and inhibiting the 5α-reductase enzyme in fibroblasts [3,9,208], and (g) binding to estrogen receptor β in the keratinocytes in the epidermis and fibroblasts in the dermis [3,9,13,16,204,205,206,207,208].

Additionally, while it is beyond the scope of this review, vitamin D has pleiotropic effects on the skin where sunlight exposure is the primary source of vitamin D for most people. Several factors influence vitamin D synthesis including latitude, season, time of day, degree of skin pigmentation, age, amount of exposure to sunlight, and sunscreen use [209]. In fact, vitamin D acts more like a hormone than a vitamin since it regulates epidermal proliferation and differentiation, protects the skin from photo-damage, and enhances wound healing [210]. Lastly, vitamin D production in the skin is associated with a complex system of steroidogenic enzymes that include neuroendocrine functions and also it is known to regulate the immune system of the skin where it has a role in certain inflammatory dermal conditions such as psoriasis and atopic dermatitis. All of these topics have been extensively reviewed elsewhere [211,212,213,214,215].

## 11. Human Microbiome—History and Background

The microbiome is a broad term defined by the ecological communities for bacteria, fungi, viruses, archaea, and mites found in and on the mouth, gut, vagina, and skin of the human body [216,217]. The term “microbiome” has been used commonly in microbiology for at least 50 years and has recently gained more recognition as the relationship between the microbiome and health became apparent via research investigations [218]. From 2007 to 2016, the National Institute of Health sponsored a collaborative, two-phased project called the Human Microbiome Project (HMP). The objective was to create opportunities for increased human health by understanding the human microbiome and its impact on health. The aim of the first phase was to identify and characterize the human microbiome. The second phase aimed to investigate the relationship between human flora and disease. The research done during this project has led to 650 papers published by the end of 2017 and with more to be published in 2018 [219].

Previously, much of the knowledge about the microbiome came from research using culture-based techniques that likely excluded uncultivable bacteria [220]. Later research used gene sequencing techniques that allowed for the detection of more diversity than culture methods. Most recently, shotgun metagenomics sequencing has been used, which allowed for sequencing of all genomic material present in a sample including human, bacterial, fungal, archaeal, and viral [221].

### 11.1. The Microbiome, Skin, and Dermal Disorders

The relationship between the gut microbiome and skin and human health through the “skin-gut” axis is better understood [222] while less is known about the effect of the skin microbiome on skin and human health. The skin is the largest organ of the body and controls water and nutrient loss while also preventing infection or invasion of harmful substances into the body. Microbes that inhabit the skin serve to protect against invading pathogens, influence or alter the immune system, and break down natural products [223,224,225]. Established at birth, the skin microbiome is hosted in the moist, dry, and oily or sebaceous regions of the body [226,227]. The regions on the skin, chemical attributes of the skin, gender, geographical location, ethnicity, depth within the epidermis, antibiotic treatment and vaccination, use of cosmetics, age, and health status influence the numbers and diversity of the skin microbiome [228].

When bacteria are in eubiosis, which means that they are in balance, commensal bacteria are harmless and potentially benefit the host [229,230]. Skin commensal bacteria can inhibit the growth of pathogens by competing with pathogens for space and nutrients, producing antimicrobial peptides (AMPs) and bactericidal compounds that restrict the growth of competitors, and inhibiting *S. aureus* biofilm production [231,232,233]. Microbial populations also serve to enhance host innate immunity by decreasing inflammation after injury and strengthening the epidermal barrier [234,235].

The types of bacteria that reside on regions of the body varies based on both the individual and the region of the body [236]. The acid mantle on the surface of the skin formed by sebum and sweat creates a slightly acidic barrier and controls the types of bacteria that live on the skin surface [237]. The density of eccrine sweat glands also influences the diversity on the skin based on the production of AMPs [238,239,240].

Keratinocytes, which are the predominate cells in the epidermis, are the first participants in the skin immune response since they use pattern recognition receptors (PRRs) to sense microbes. PRRs can increase the expression of AMPS, cytokines, and chemokines, which creates an antimicrobial effect as well as recruitment and modulation of other immune cells [241]. The understanding of the role of Langerhans cells (LCs), which are dendritic cells of the skin, is currently growing and changing. LCs not only participate in immune surveillance and homeostasis but also affect disease by either inducing tolerance or mediating inflammation [242]. 

Dysbiosis occurs when the microbial community is altered or impaired compared with normal conditions, which creates the opportunity for skin conditions or diseases to develop. Acne, which is a common skin condition that affects approximately 85% of adolescents and young adults, has been associated with the overgrowth of *Propionibacterium acnes* (*P. acnes*) and specifically with certain strains of the bacteria [243,244,245]. Even though psoriasis patients have microbiota distinct from healthy, unaffected skin, the direct link between the skin microbiota and the disease remains unclear [246]. Individuals with atopic dermatitis (AD) showed an increase in *Staphylococcus aureus* (*S. aureus*) during flares along with a decrease in microbial diversity [247]. After AD treatments, the bacterial diversity increased, which suggests a relationship between microbial diversity and improvements in disease activity [245]. The relationship between skin dysbiosis and epidermolysis bullosa, rosacea, and blepharitis has also been noted [248,249,250].

In this regard, it is notable that the hair follicle (infundibulum and isthmus) is particularly vulnerable to the invasion of micro-organisms that cause hair follicle inflammation and other skin pathologies when the major histocompatibility complex (MHC) class-I-dependent immune mechanism is altered [251,252,253]. In a series of journal reports, the immunology of the hair follicle has been studied where the atypical expression of MHC I is associated with altered immune recognition. However, it has also been shown that MHC class-I expression can be normalized through treatment with α-MSH, IGF-I, or TGF-β 1 to restore immune privilege in clinically desired conditions like alopecia [252]. 

### 11.2. The Microbiome, Inflammation, Chemoprotection, and the Future

While there is some understanding of the relationship between the microbiome and inflammatory diseases such as psoriasis and acne discussed previously, research on how the skin microbiome is related to cancer is in its infancy. However, there is potential of utilizing microbiome in the prevention, earlier diagnosis, and the treatment of skin cancers [254]. Microbial exposure plays a critical role in suppressing cancer-promoting inflammation by strengthening the network between regulatory T cells (T-regs) and both T-helper (Th17) and inflammatory cytokine interleukin-23 (IL-23) [255]. Toll-like receptors (TLRs) induce inflammatory responses against invading pathogens. However, unregulated TLR activation leads to chronic inflammation that may lead to skin cancer [256]. Research has shown how microbial components heighten immune surveillance that provide anti-tumor activity in the bladder and colon [257,258]. Additionally, workers who are exposed to environmental microbiota such as endotoxins and cotton dust have been reported to have lower cancer rates, which suggests a relationship between the microbiome and cancer [259]. 

As our understanding of the relationship between the skin microbiome and disease increases, alterations of the skin flora have a potential role for treating certain diseases. For example, the use of probiotics, live bacteria that benefit the host, and prebiotics known as “food” for the good bacteria has been established in gut health and continues to be investigated for skin health [260,261]. *Staphylococcus epidermidis* (*S. epidermidis*), which is a commensal skin bacterium, can ferment glycerol and create inhibition zones to repel a colony of overgrown *P. acnes* [262]. In a mouse model, sucrose applied topically showed an increase in commensal *S. epidermidis* and a decrease in pathogenic *P. acnes*, which suggests that there is prebiotic potential [263]. It has also been shown that homeostasis can be restored in mice with AD-affected skin by the transplant of normal skin microbiota [264]. 

Altering the gut microbiome to prevent and treat disease has been established while the effect of skin microbiome on disease is still a developing area of research that needs further exploration. Current research examines the existing states of the skin microbiome in healthy and diseased populations, but little research has been performed to explore the effect of specific oral compounds on skin health through alterations of the skin microbiome. It may be fruitful for future research to examine the therapeutic potential of the modulation of the skin microbiome by oral and topical compounds including those reviewed in this article.

## 12. Conclusions

Since the year 2000, oral administration of natural molecules and minerals have been reported to improve skin health from the inside layer to the dermal and outer structural and functional skin layers involved in maintaining homeostasis and providing protection against photo-aging via UV-induced damage of DNA, apoptosis, and mutations [13,90,203]. The concept of nutri-cosmetics is a novel and applicable term for this mode of administration [203]. In addition, the strong antioxidant properties for many of the natural compounds that have diverse biological and molecular activities guard against the inflammatory response, ROS production, skin pigmentation, dermal wrinkles, and enhancing skin barrier properties especially of the stratum corneum. The recent emphasis on the skin microbiome highlights the discoveries of potential interactions of microbiota diversity (that change with age, gender, geographical location) with dermal structural and functional properties that were previously unknown [216,217,218,222,265]. Future studies will undoubtedly reveal the complex interactions of the skin microbiome with dermal health and skin aging.

## Figures and Tables

**Table 1:** Summary of all natural compounds and minerals covered in this review.

Natural Compound or Mineral	Mechanism of Action(s) Involved in Maintaining Skin Health
1. Collagen (Section 2)	building block of collagen and elastin fibers-improves skin and nail health; inhibits matrix metalloproteinases (MMPs); stimulates fibroblast function
2. Ceramides (Section 3)	provides the major component of the lipid “mortar” of the stratum corneum essential in the structure and maintenance of skin barrierintegrity; also involved in cell proliferation, differentiation and apoptosis
3. Beta Carotene (Section 4)	provitamin A molecule, acts as an antioxidant, anti-inflammatory agent and blocks ROS formation and/or ability to quench free radicals; prevents cellular damage, premature skin aging and skin cancer
4. Astaxanthin (Section 5)	potent antioxidant; anti-inflammatory agent; prevents DNA damage & enhancemitochondrial function, provides UV protection; activates the Nrf2 pathway toto stimulate production of other antioxidants; inhibits MMPs; stimulates collagenproduction and wound healing
5. Coenzyme Q10 (Section 6)	antioxidant; anti-aging properties-enhances collagen; potential treatment for psoriasis; accelerates generation of ATP levels after irradiation of fibroblasts
6. Colostrum (Section 7)	contains, growth factors and other immune regulatory factors that promote growth of keratinocytes and wound healing
7. Zinc (Section 8)	importance for skin morphogenesis, repair and maintenance such as wound healing
8. Selenium (Section 9)	acts as a cofactor for glutathione peroxidase (GPX) removing harmful peroxides; involved in DNA synthesis and repair; prevents oxidative stress and UVB-radiation; also acts as an antioxidant

## Abbreviations

Da	Dalton
kDa	kilo Dalton
g	gram
kg	kilogram
L	Liter
mol	mole
mg	milligram
ppm	parts per million
μg	microgram
μmol	micro mole
μM	micro molar
^3^H	radioactive hydrogen (tritium)
^14^C	radioactive carbon 14
5α-DHT	5α-dihydrotestosterone
5α-Reductase	Steroid enzyme that converts testosterone into 5α-DHT
AD	Atopic dermatitis
Akt	Protein kinase B (a serine/threonine-specific protein kinase)
ALA	Alanine
α-MSH	α-melanocyte stimulating hormone
AMP	Antimicrobial peptides
AP-1	Activator protein 1
ATP	adenosine triphosphate
BPH	Benign prostatic hyperplasia
COX	Cyclo-oxygenase
DNA	Deoxyribonucleic acid
EGF	Epidermal growth factor
ERβ	Estrogen receptor β
FGF	Fibroblast growth factor
GI	Gastrointestinal tract
G2	Gap 2 phase (cell cycle)
Glc-Cer	Glucosylceramide
Gly	Glycine
GPX	Glutathione peroxidase
HaCaT	Human keratinoctyes
Hyp	Hydroxy proline
HMP	Human microbiome project
HDL	High-density lipoprotein
HO-1	Heme oxygenase 1
IL	interleukins
IgA, IgG, IgM	Immunoglobulin A, G, or M
IGF	Insulin-like growth factor
KOD	36. amino acid synthetic collagen. The sequence of the peptide is (Pro-Lys-Gly) (Pro-Hyp-Gly) (Asp-Hyp-Gly), and in single-letter amino acid abbreviation is (P-K-G); (P-O-G) (D-O-G), giving it the name KOD
LC	Langerhans cells
LDL	Low-density lipoproteins
MCH	Major histocompatibility complex
MDA	Malondialdehyde
MMP	Matrix metalloproteinases
NF-κB	Nuclear factor κ-light-chain-enhancer of activated B cells
Na_2_SeO_3_	Sodium selenite
Nrf2	Nuclear factor (erythroid-derived 2)-like 2 (basic leucine zipper)
PDGF	Platelet derived-growth factor
Pro	Proline
PRR	Pattern recognition receptor
RDA	Recommended dietary allowance
SEPW1	Seleno-protein 1
RNA	Ribonucleic acid
ROS	Reactive oxygen species (oxidative stress)
RSSC	Residual skin surface components
SeMet	Seleno-L-methionine
Ser	Serine
SIRT	NAD-dependent deacetylase sirtuin-1
TEWL	Trans-epidermal water loss
TGF	Transforming growth factor
Th17	Helper T cell 17
TMP	Tissue inhibitor of matrix metalloproteinases
TLR	Toll-like receptors
TNF-α	Tumor necrosis factor α
T-reg	Regulator T cells
US	United States
USA	United States of America
USFDA	United States Federal Drug Administration
UV	Ultraviolet
ZIP2	Zinc transporter-2
